# Association of the barriers of pharmaceutical care perceived by clinical pharmacists and occupational stress in tertiary hospitals of China

**DOI:** 10.3389/fpubh.2024.1342565

**Published:** 2024-04-09

**Authors:** Yu Zhang, Yuankai Huang, Xiaoyu Xi

**Affiliations:** National Medical Products Administration Key Laboratory for Drug Regulatory Innovation and Evaluation, China Pharmaceutical University, Nanjing, China

**Keywords:** clinical pharmacist, occupational stress, barriers, pharmaceutical care, clinical pharmacy

## Abstract

**Objective:**

As an important member of the healthcare team, clinical pharmacists’ occupational stress will lead to a decline in the quality of pharmaceutical care. According to person-environment fit theory, barriers of pharmaceutical care perceived by clinical pharmacists may be a potential factor influencing occupational stress. This study aimed to assess the association between the specific barriers of pharmaceutical care perceived by clinical pharmacists and their occupational stress in China.

**Method:**

A field-based questionnaire survey of tertiary hospitals was conducted in 31 provincial administrative regions in mainland China using a multi-stage stratified sampling method. Data on occupational stress, barriers of pharmaceutical care perceived by clinical pharmacists and other factors of job stress were collected using the Brief Job Stress Questionnaire and a self-administered instrument. The instruments have undergone multiple rounds of pilot investigations, and their reliability is acceptable. Ordinary least squares regression was used to evaluate the association of the perceived barriers and other factors with their occupational stress.

**Result:**

A total of 625 clinical pharmacists from 311 tertiary hospitals in China (response rate = 84%) participated. Perceived resource dimension barriers (*p* = 0.00) and self-improvement dimension barriers (*p* = 0.01) were associated with increased occupational stress of the participants. In addition, clinical pharmacists with senior professional titles and engaged in neurology and ICU have higher occupational stress.

**Conclusion:**

By removing barriers to pharmacists’ resources and self-improvement, it is possible to better meet the work needs of clinical pharmacists and may effectively reduce occupational stress, thereby improving the quality of pharmaceutical services.

## 1 Introduction

Occupational (job) stress is defined as the tension or repression employees feel in their work (1). The current mainstream view is that occupational stress is the result of the interaction between personal characteristics and environmental stimuli, emphasizing that stress is a process (2). Robbins’ stress model, which posits a causal relationship between stressors, experienced stress, and stress outcomes, has been widely accepted by researchers. Stressors can be classified into environmental, organizational, and personal factors. These three stressors and individual differences interact to produce stressful experiences. These experiences can lead to physical, psychological, and behavioral symptoms (3).

Among the unique members of the healthcare team, clinical pharmacists play an essential role in ensuring rational drug use (4). Clinical pharmacists under considerable stress in China (5–7). Common stressors include lack of collaboration between physicians and pharmacists, insufficient information about patients’ conditions, and lack of clinical therapeutic knowledge (8–11). However, excessive occupational stress will damage the physical and psychological health of pharmacists, resulting in higher turnover rates or false presenteeism, lower work efficiency and, ultimately, higher incidence of medical malpractice (12–15).

**Figure 1:**
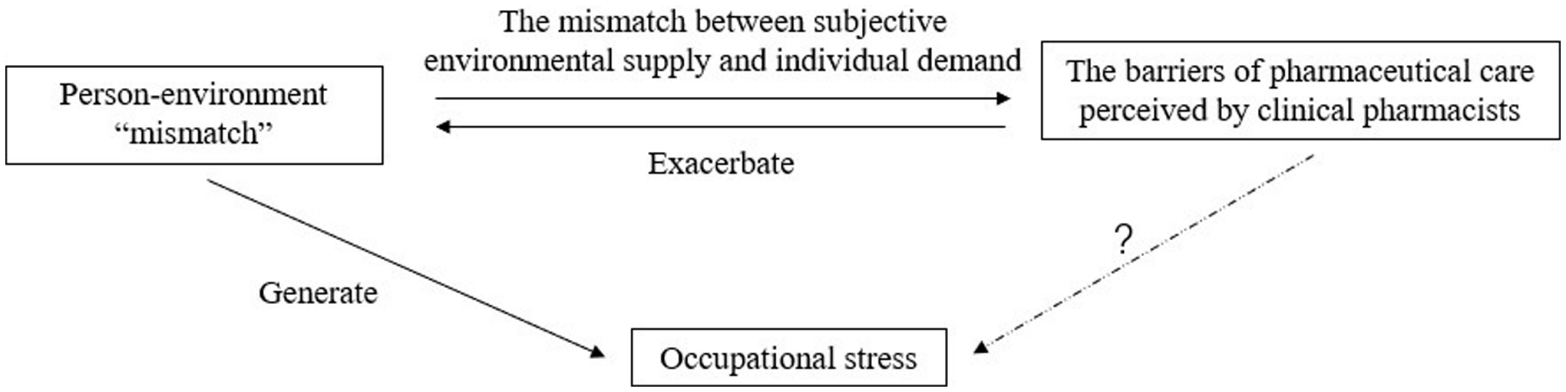
Conceptual model.

The barriers of pharmaceutical care are the factors that hinder or negatively affect the work of clinical pharmacists in the process of implementing pharmacy services. Hospitals around the world face barriers to pharmaceutical care to varying degrees (7). Pharmaceutical care in Chinese hospitals is in the development stage, and the barriers are various and complex, such as the professional level of pharmacists is uneven, the business scope of pharmacists is not refined, pharmacists do not have the corresponding value return, and equipment and facilities have not yet met the service requirements, which cause the development dilemma of pharmaceutical care (7). Person-environment fit theory is one of the classic theories concerning job-related stress and has been widely applied in studies of occupational stress among health care providers. It describes the theoretical relationship between the stress and stressors as a “misfit” or “mismatch” between the individual (personal demands and capabilities) and the environment (environmental demands and supply) as being one of the main causes of occupational stress (16–19). The various barriers perceived by clinical pharmacists in the process of providing pharmaceutical care are mainly a manifestation of the mismatch between subjective environmental supply and individual demand. The creation of these barriers will exacerbate the mismatch between the individual and the environment, which may lead to the creation of occupational stress, i.e., the barriers to pharmaceutical care may be a potential factor influencing occupational stress. The conceptual model can be seen in Figure 1.

At present, there are many related studies on occupational stress of clinical pharmacists in foreign countries, including stress status, causes, results of pressure, mechanism of action model and so on. However, there are some problems such as old age and outdated data. In addition, due to the late establishment of the work system of clinical pharmacists in China, relevant research in China is relatively rare, and there is still a lack of comprehensive and systematic research on the occupational stress of clinical pharmacists (7). The main ways of reducing pharmacists’ occupational stress are psychological intervention, increasing salaries, increasing social support, and providing learning and training opportunities. The above theory suggests that the barriers of pharmaceutical care may be a potential factor affecting occupational stress. However, there is no direct evidence to show whether it affects occupational stress in clinical practice. Moreover, due to the complexity of pharmaceutical care barriers in China, the existing evidence cannot clearly reflect the influence of different barriers on the occupational stress of clinical pharmacists. Therefore, it is of great significance to clarify which specific barriers will affect the barriers of pharmaceutical care of clinical pharmacists.

This study aimed to assess the association between the barriers of pharmaceutical care perceived by clinical pharmacists and occupational stress, and to consider the reduction of barriers to pharmaceutical care by clinical pharmacists in order to reduce occupational stress in clinical pharmacists and improve the quality and efficiency of clinical pharmaceutical care. This study only considered pharmaceutical services in tertiary hospitals. This is due to the 3-tier hierarchical structure of the healthcare system in China. Clinical pharmacy services are mainly provided in tertiary hospitals, which account for more than half of the health care burden in China (20). This study conducted an empirical survey of clinical pharmacists in tertiary hospitals in China to clarify the quantitative relationship between their subjective perceived barriers of pharmaceutical care and occupational stress. Based on the findings, recommendations are provided to reduce the occupational stress of Chinese clinical pharmacists. This study may also be of value to other developing or underdeveloped countries.

## 2 Methods

### 2.1 Study design and participants

A stratified sampling strategy was adopted to investigate the occupational stress and subjective perceived the barriers of pharmaceutical care of clinical pharmacists in tertiary hospitals in China from July to August 2019. The inclusion and exclusion criteria were as follows. The inclusion criteria: ①full-time clinical pharmacists of the sampled hospitals; ②available and willing to participate in the study by completing the questionnaire; ③able to sign the informed consent document. The exclusion criteria: clinical pharmacists in training (students on clerkships or internships) and visiting clinical pharmacists were excluded (20).

First, all 31 provincial administrative regions (including provinces, autonomous regions, and municipalities directly under the central government) in mainland China were covered in the sampling (21). Next, cities in each province, autonomous region or district in each municipality were evenly divided into 3 groups according to their 2018 *per capita* gross domestic product, which is associated with the factors influencing the working status of medical staffs, such as the quality of health care techniques and need and utilization of health care thereby generating 93 groups. Within each group, 1 city or district was selected using the random number method; thus, 93 cities or districts were selected. In each selected city or district, 2 to 4 tertiary hospitals were surveyed by convenience based on the hospital administrators’ permission to conduct the survey, with the hospital level verified by consulting the hospital information tool by the National Health Commission of China. This ensured that at least 186 hospitals would be selected. In each surveyed hospital, 2 participants were recommended by the hospital administrator(s) or another participant who completed the survey. Overall, 744 questionnaires were distributed. The distributions of sex, age, technical titles, and hospital type of the sample were under real-time monitoring, according to which the last stage of sampling was manipulated to make the distribution of the above-mentioned attributes of the sample relatively consistent with the attributes of the population of clinical pharmacists in China reported previously.

### 2.2 Instrument

An expert panel of 2 administrators and 2 teaching clinical pharmacists from tertiary hospitals, together with 3 experts in clinical pharmacy education from universities, were consulted for the design of the study questionnaire. The questionnaire comprised the following 3 sections: basic information, occupational stress and the barriers of pharmaceutical care perceived by clinical pharmacists.

Basic Information of Interviewees: Sex, age, marital status and years of practice, technical title, specialized field and types of hospital were included, because these factors are potentially associated with the health care providers’ occupational stress in China (22–24).

Occupational stress: The instrument Brief Job Stress Questionnaire (BJSQ) scale was used as the measure of occupational stress in this study. This questionnaire includes 15 items on 3 dimensions, job demand, job control, and support, using 4-point Likert items (1 = agree; 2 = somewhat agree; 3 = somewhat; 4 = disagree). The items in the first dimension of the scale were reversely scored, with higher scores meaning more serious occupational stress. The BJSQ was originally developed and validated in Japan, where it has been widely used and established as a method for assessing job stress in Japan. And there have been studies using the BJSQ questionnaire scores to conduct regression analysis on the factors affecting the occupational stress of medical workers (25, 26). Japan has a similar cultural background and environment as China. The BJSQ was originally in English and translated by a native speaker of English who is fluent in Chinese and a Chinese translator proficient in Chinese-English translation separately. The 2 versions were reviewed, synthesized, and revised by the 2 translators and the expert panel until they reached a consensus on all translations. In the original questionnaire, the 16th item only plays a role in the verification of questionnaire development, and will be used separately as a variable to participate in the robustness test in this study.

The barriers of pharmaceutical care perceived by clinical pharmacists: There are a large number of instruments measuring the barriers of hospital pharmaceutical care, but most of them are developed in foreign countries, and some of them have been systematically designed in terms of dimensions and items, which have been applied to local large sample empirical studies and have certain reference value. However, there are still some shortcomings, which may not be suitable for the hospital pharmaceutical care environment in China (7). Therefore, through literature research and expert survey, the study developed an instrument for the barriers of pharmaceutical care perceived by clinical pharmacists to be used. The Chinese literature search database CNKI; Wanfang; VIP and the English database Medline; ScienceDirect were searched, using the following general search strategy: (“Hospital” OR “Pharmaceutical Care” OR “Pharmacy Service”) AND (“Barrier” OR “Obstacle”). The instruments were compared in terms of development background, applicable object, development process and measurement content. A total of 9 researches were included. According to the literature summary, the possible barriers of pharmaceutical care for clinical pharmacists were obtained, and the item pool was formed and translated. Then, the item pool was screened through the expert group interview, and the repeated items were screened out to form the first draft of the scale. Each item was provided in the form of its complete name or description and presented to participants as, “Have you encountered any of the following obstacles in your work?” with 18 items in total and a response of “Yes (1)”or “No (0).”

Pre-test: All instruments were composed in Chinese and proofed by the expert panel and researchers, followed by a multi-round pilot survey in tertiary hospitals in Nanjing, Jiangsu Province, China, to test the rationality, comprehension and readability of the questionnaire. The pilot participants for each round met the general inclusion criteria for the study. After each round, revisions were made according to the feedback. The pilot process continued until no new feedback was generated. Finally, 4 rounds of pilot testing were completed and the draft was finalized. A pre-test of the questionnaire was then conducted in April 2019 among 47 clinical pharmacists from 24 tertiary hospitals in 6 cities of Jiangsu Province, China, using convenience sampling. The reliability of the BJSQ and the knowledge and skills instrument were tested using Cronbach’s alpha. The reliability of both instruments was acceptable (Cronbach’s alpha 0.76 and 0.80, respectively). The final questionnaire is available online as Appendix 1.

### 2.3 Data collection

A total of 46 undergraduate students majoring in general pharmacy or clinical pharmacy were recruited as data collectors and trained with the procedure of accessing the potential participant and conducting the survey and the standardized explanations for potential questions from the participants. Every 2 data collectors investigated 1 set of geographically neighboring cities or districts in pairs during July and August of 2019. After obtaining the hospital administrators’ consent, during the noon break of the hospital, the data collectors asked the potential participants for their basic information to determine whether they meet the study inclusion criteria and then provided the eligible participants with the purposes, contents, and requirements of the survey and checked their willingness to participate again. Those who were willing to participate signed the consent form and decided the time and an undisturbed place for survey with the data collectors.

Using an online survey system on mobile phones or tablet computers, the data collectors orally interviewed the participants with each item of the questionnaire and recorded their responses, and then, the survey system converted the data into electronic documents. The data collectors were not allowed to provide any view on the questionnaire, but only the requirements or instructions of questionnaire filling. The survey system allowed the users to set restrictions on format of responses and ensured the quality of the data. A total of 5 postgraduates were recruited and trained to review the uploaded documents and immediately return those with data entry errors or damaged data, which were corrected through return visits by data collectors when possible (21).

### 2.4 Data analysis

The dependent variable of this study was the BJSQ score of clinical pharmacists, the independent variable was the barriers of pharmaceutical care perceived by clinical pharmacists, and the covariates included sex, age, marital status and years of practice, technical title, specialized field and types of hospital. Since the barriers of pharmaceutical care scale was newly developed, exploratory factor analysis (EFA) was adopted to reduce its dimension and confirmatory factor analysis (CFA) was performed. The weight coefficients of each item were calculated using Principal Component Analysis (PCA) (27). Descriptive statistics were used to report the characteristics of the sample. Mean and standard deviation were used in the statistical description of continuous variables, and percentage was used in the statistical description of categorical variables. Ordinary least squares regression was used to assess the association of each independent variable with occupational stress. Multicollinearity was assessed by examining the variance inflation factor (VIF). An independent variable with a VIF greater than 10 indicates that it is collinear with the other independent variables and should be removed. The VIF was examined again when an independent variable with the highest VIF greater than 10 was removed. This was repeated until multicollinearity was suspected.

To assess the robustness of the results, clinical pharmacists’ job satisfaction was included in the regression model as a covariate to check whether the magnitude, direction and *p*-value of the regression coefficients of the respective variables in the regression model were significantly different from the original model. Microsoft Excel 2016 was used for data cleaning, calculations and descriptive statistics. EFA and CFA were carried out using SPSS 24 and Amos. Stata 15.0 was used for data analysis.

## 3 Results

Overall, 625 questionnaires from 311 tertiary hospitals of 744 deployed questionnaires were uploaded and completed (response rate = 84%). The main participant characteristics are found in Table 1. The mean age of the participants was 35.06 years (SD = 6.44). Among the participants, approximately two-thirds were of female sex (65.6%) and most were married (85.1%). And their mean years of practicing as a clinical pharmacist was 9.3 years (SD = 6.62). 30.6% of participants had junior technical titles and 56.0% had intermediate title. (In China, clinical pharmacist titles are classified into junior, intermediate, and senior levels). Furthermore, most of them were working in general hospitals (75.5%). The clinical pharmacists were mainly engaged in anti-infectives (23.7%), cardiology (16.3%), respiratory medicine (14.2%) and gastroenterology (13.3%). The sample has a similar distribution of sex, age, education background, and technical titles to those indicators of the sample reported in a national study of clinical pharmacists in China in 2015, indicating acceptable representativeness of the sample (28).

**Table 1:** Clinical pharmacist sample information.

Item	N(%)
Age, mean(SD)	35.06 (6.4)
Years of practicing as a clinical pharmacist, mean (SD)	9.3 (6.6)
Sex, n (%)	
Male	215 (34.4%)
Female	410 (65.6%)
Marital status, n (%)	
Unmarried	89 (14.2%)
Married	532 (85.1%)
Other (divorced, widowed, etc.)	4 (0.6%)
Technical titles, n (%)	
Junior title	191 (30.6%)
Intermediate title	350 (56.0%)
Deputy senior title	72 (11.5%)
Senior title	12 (1.9%)
Specialized field the clinical pharmacist engaged in^a^	
Anti-infectives	148 (23.7%)
Cardiology	102 (16.3%)
Respiratory medicine	89 (14.2%)
Gastroenterology	83 (13.3%)
Nephrology	32 (5.1%)
Oncology	61 (9.8%)
Organ transplantation	8 (1.3%)
Intensive care	39 (6.2%)
Endocrinology	48 (7.7%)
Neurology	43 (6.9%)
Other	133 (21.3%)
Type of hospital, n (%)	
General hospital	472 (75.5%)
Specialized hospital	33 (5.3%)
Traditional Chinese medicine hospital	82 (13.1%)
Other	38 (6.1%)

^a^Some clinical pharmacists are engaged in two or more specialties, the sum of this percentage is not 100%.

For the questionnaire of the barriers of pharmaceutical care perceived by clinical pharmacists, this study reduced its dimensions into four dimensions through EFA and deleted three questions. Then CFA was carried out, and the model fit index was good, and the standardized factor loading was all greater than 0.4, and the model was acceptable (full details are provided in Appendix 2). The standardization Cronbach’s Alpha was used for reliability test. The Kaiser-Meyer-Olkin Measure of Sampling Adequacy and Bartlett’s Test of Sphericity were used to conduct the validity test. The reliability and validity test results of each dimension of work disability after dimensionality reduction can be seen in Table 2. The results showed that Cronbach’s Alpha of all dimensions was greater than 0.5, indicating acceptable reliability. Kaiser-Meyer-Olkin measures of Sampling Adequacy were all greater than 0.5, and the Bartlett’s Test of Sphericity results were statistically significant, and the validity was acceptable.

**Table 2:** Reliability and validity test results of each dimension of the barriers of pharmaceutical care questionnaire.

Dimension	Items	Reliability	Validity			
		Cronbach’s alpha	Kaiser-Meyer-Olkin measure of sampling adequacy	Bartlett’s test of sphericity		
				Approx. chi-square	df	Sig.
Resources	6	0.802	0.827	1025.063	15	0.000
Cooperation	3	0.832	0.704	747.573	3	0.000
Leadership support and pharmacist’s rights	4	0.712	0.706	543.622	6	0.000
Self-improvement	2	0.688	0.500	200.038	1	0.000

Table 3 shows the respondents’ perception of various the barriers of pharmaceutical care after dimension reduction and the weight coefficient calculated according to PCA.

**Table 3:** The perception of the barriers of pharmaceutical care.

Items	N(%)	Weight coefficient
Resources (6)		
Lack of electronic systems	182 (29.1%)	0.014
Electronic systems are difficult to use	155 (24.8%)	0.035
Standardized procedures and records	169 (27.0%)	0.056
Staffing of pharmacy	276 (44.2%)	0.076
Specific place	175 (28.0%)	0.030
Specific time	216 (34.6%)	0.044
Cooperation (3)		
Physician’s communication and support	121 (19.4%)	0.078
Other staff’s support and communication	136 (21.8%)	0.080
Patient’s communication and support	143 (22.9%)	0.084
Leadership support and pharmacist’s rights (4)		
Support from leaders of hospitals	127 (20.3%)	0.052
Support from department leaders	70 (11.2%)	0.119
Support from the legal system	204 (32.6%)	0.119
Patient’s medical information	51 (8.2%)	0.071
Self-improvement (2)		
Opportunities for continuing education	123 (19.7%)	0.075
Time for continuing education	182 (29.1%)	0.068

The results of the regression analysis are provided in Table 4. Perceived resource dimension (coef. = 10.70, *p* = 0.00, 95%CI = [5.00, 16.41]) and self-improvement dimension barriers (coef. = 6.71, *p* = 0.01, 95%CI = [1.97, 11.46]) had higher occupational stress than those who did not perceive these two dimensions of barriers. In the covariable, clinical pharmacists with senior title (coef. = 2.99, *p* = 0.06, 95% CI = [0.17, 6.16]) and who majored in ICU (coef. = 1.46, *p* = 0.05, 95%CI = [0.01, 2.91]) and neurology (coef. = 1.06, *p* = 0.09, 95%CI = [−0.16, 2.27]) had higher occupational stress.

**Table 4:** Regression analysis of factors associated with occupational stress.

Items	M1	M2
Model *p* value	< 0.001	< 0.001
Adjusted R^2^	0.1597	0.2614

Item	Original result			Robustness test		
	coef.	95% CI	*p* value	coef.	95% CI	*p* value
Perceived (ref = no such barrier)						
Resources	10.70	[5.00,16.41]	0.00***	6.99	[1.73,12.26]	0.01**
Cooperation	0.46	[−4.56,5.47]	0.86	−0.23	[−5.12,4.65]	0.93
Leadership support and pharmacist’s rights	3.90	[−2.98,10.78]	0.27	2.24	[−4.35,8.83]	0.51
Self-improvement	6.71	[1.97,11.46]	0.01**	5.28	[0.89,9.67]	0.02**
Sex						
Female (ref = male)	0.39	[−0.33,1.12]	0.29	0.42	[−0.28,1.11]	0.24
Age	0.05	[−0.04,0.14]	0.30	0.04	[−0.05,0.12]	0.37
Marital status (ref = unmarried)						
Married	−1.37	[−2.47,-0.27]	0.02**	−1.24	[−2.28,-0.19]	0.02**
Other (divorced, widowed, etc.)	4.10	[−4.42,12.62]	0.35	3.59	[−3.88,11.06]	0.35
Years of practicing as a clinical pharmacist	−0.04	[−0.13,0.04]	0.33	−0.03	[−0.11,0.05]	0.48
Technical titles (ref = junior title)						
Intermediate title	0.41	[−0.46,1.28]	0.36	0.45	[−0.36,1.26]	0.27
Deputy senior title	0.19	[−1.26,1.64]	0.80	0.20	[−1.15,1.55]	0.77
Senior title	2.99	[−0.17,6.16]	0.06*	2.98	[0.19,5.77]	0.04**
Specialized field the clinical pharmacist engaged in (ref = unengaged)						
Anti-infectives	0.53	[−0.36,1.41]	0.24	0.60	[−0.25,1.45]	0.17
Cardiology	0.02	[−0.92,0.95]	0.97	0.14	[−0.75,1.03]	0.76
Respiratory medicine	0.71	[−0.36,1.79]	0.19	0.86	[−0.15,1.88]	0.09
Gastroenterology	−0.24	[−1.38,0.90]	0.68	0.01	[−1.09,1.12]	0.98
Nephrology	−0.59	[−2.11,0.93]	0.45	−0.62	[−1.99,0.75]	0.38
Oncology	0.61	[−0.66,1.88]	0.35	0.63	[−0.58,1.83]	0.31
Organ transplantation	−1.70	[−4.26,0.87]	0.19	−2.30	[−5.47,0.87]	0.15
Intensive care	1.46	[0.01,2.91]	0.05*	1.32	[−0.08,2.72]	0.07*
Endocrinology	0.09	[−1.29,1.48]	0.89	−0.20	[−1.54,1.15]	0.77
Neurology	1.06	[−0.16,2.27]	0.09*	1.04	[−0.08,2.15]	0.07*
Other	−0.68	[−1.74,0.37]	0.20	−0.20	[−1.21,0.80]	0.69
Type of hospital (ref = general hospital)						
Specialized hospital	−1.44	[−2.74,-0.14]	0.03**	−1.73	[−3.02,-0.44]	0.01**
Traditional Chinese medicine hospital	0.03	[−0.97,1.03]	0.95	0.17	[−0.75,1.09]	0.72
Other	0.31	[−2.19,2.81]	0.81	−0.09	[−2.11,1.93]	0.93
Constant term	34.32	[31.47,37.18]	0.00	32.83	[30.20,35.46]	0.00***
Satisfied with my job (ref = agree)						
Somewhat agree				2.83	[2.10,3.55]	0.00***
Somewhat disagree				5.28	[3.58,6.98]	0.00***
Disagree				2.66	[−2.98,8.29]	0.36

**p < 0.1; **p < 0.05; ***p < 0.01.

## 4 Discussion

Pharmaceutical services are transitioning from a focus on drugs and diseases to a focus on patients and their health. Clinical pharmacists are now highly regarded as key players in ensuring the appropriate use of medications. Based on the person-environment fit theory, this study theoretically hypothesized and tested the association between the barriers of pharmaceutical care perceived by clinical pharmacists and occupational stress in tertiary hospitals of China. This study presents the first quantitative investigation into the correlation between perceived barriers to pharmaceutical care among clinical pharmacists and occupational stress. While previous research on occupational stress in healthcare professionals has primarily focused on doctors and nurses, studies on pharmacists’ occupational stress have mainly examined the outcomes of stress, such as its impact on pharmacists’ quality of life and job satisfaction, and its contribution to occupational burnout (29–31). There is a scarcity of quantitative research on effective methods for reducing occupational stress. It is the first time to reduce occupational stress from the perspective of reducing the barriers of pharmaceutical care perceived by clinical pharmacists, which provides a new way to reduce occupational stress of clinical pharmacists. The results showed that the perceived barriers of resource dimension and self-improvement dimension were associated with the increased occupational stress among clinical pharmacists from tertiary hospitals in China. In addition, their technical titles and specialized field they engaged in were associated with their occupational stress.

For the perceived barriers of resource dimension, firstly, modern equipment and technology are increasingly used in hospital information management and service systems (32, 33). However, most of the hospital information system application software products in the market mainly focus on financial and administrative management, while the clinical practice function is relatively less. Taking rational drug use software as an example, although there are several mainstream software in the market, they still have certain shortcomings in various aspects, such as not enough clinical connection, poor intelligence, update lag, and so on, which cannot fully meet the work demand of clinical pharmacists (34, 35). And clinical pharmacists without good training may have problems in the operation process, which makes it difficult for them to complete work tasks on time, resulting in excessive subjective perception of workload. Therefore, the lack of electronic pharmaceutical care management system or the difficulty in using the system will increase the occupational stress of clinical pharmacists. Secondly, standard working procedures are an important condition for clinical pharmacists to perform their work smoothly (36). Non-standard working procedures, such as service process and documentation records, will lead to incomplete medical information records and unimpeded communication, resulting in reduced work efficiency and increased occupational stress. The shortage of pharmacists and the lack of dedicated time and place to provide pharmaceutical care will increase the occupational stress of clinical pharmacists. This may be due to insufficient human resources and vague job responsibilities of clinical pharmacists, resulting in the need for clinical pharmacists to do a lot of work outside pharmaceutical care (37). In addition, the lack of a dedicated workplace can hinder the effective work of clinical pharmacists, in particular the efficiency and quality of prescription review, medication calendaring, adverse reaction reporting and patient information management, leading to increased occupational stress (38).

For the self-improvement barriers dimension, continuing education is an important link for clinical pharmacists to supplement their knowledge and improve their skills. Since 2000, the state has paid more attention to the training of clinical pharmacists and vigorously carried out the construction of clinical pharmacist training base. However, the content and form of continuing education are relatively simple, mainly online teaching, but its content setting is mostly theoretical, lack of on-site pharmaceutical care, drug management and other practical operation training content, training effect is poor, lack of practical training opportunities (32). In addition, the heavy workload of clinical pharmacists means that they have less free time to participate in continuing education. As a result, clinical pharmacists are unable to improve their personal skills through continuing education and to meet higher job demands. Difficulties in career development lead to increased occupational stress. In this regard, hospitals should attach importance to the continuing education of clinical pharmacists, provide regular opportunities for continuing education of clinical pharmacists, such as attending lectures, seminars, case exchange meetings, etc., and ensure their participation in continuing education time.

The influence of the cooperation dimension, leadership support and pharmacists’ rights dimension on occupational stress was not significant. This could be due to the lack of discourse power of pharmacists in diagnosis and treatment (38). Some studies have shown that Chinese physicians are not very receptive to clinical pharmacists’ suggestions, and that clinical pharmacists’ clinical involvement is not sufficient (39, 40). Therefore, the dimension of barriers to cooperation does not significantly increase occupational stress. In addition, the interest relationship within the hospital environment is complex. The drug price surcharge of public hospitals has been abolished, but the charging mechanism of hospital pharmaceutical services has not yet been formed, and the value of hospital pharmaceutical services to hospital operation is not clear. Therefore, the support from hospitals and department heads to clinical pharmacists may not be sufficient. In addition, the relevant systems and regulations for the work of clinical pharmacists in China are still being explored and established, and most of them are relatively broad and can only be used as guidelines. There is no legal protection for the right to obtain patient information, which is an important basis for clinical pharmacists to provide pharmaceutical services, indicating that the awareness of pharmacists’ rights has not yet been formed. Therefore, the barriers of leadership support and pharmacists’ rights dimension did not significantly increase occupational stress.

The relationship between other factors and occupational stress in clinical pharmacists is also worth exploring. Clinical pharmacists with senior professional titles had higher occupational stress, which could be due to the fact that they often undertake more difficult work, including attending clinical consultations and discussing difficult cases. Clinical pharmacists engaged in neurology and ICU have higher occupational stress than those in other professional fields. These two specialties require a higher level of medical knowledge and clinical skills. Patients in neurology have complex conditions and are difficult to treat and manage with medication. Intensive care unit patients are usually critically ill and the clinical problems have individual differences and interprofessional characteristics. Clinical pharmacists face the double pressure of psychological and skills, therefore the core professional knowledge of clinical pharmacists needs to be strengthened.

Overall, the dimensions of resources and self-improvement have a significant impact on pharmacists’ occupational stress. By removing barriers to pharmacists’ resources and self-improvement, it is possible to better meet pharmacists’ work needs, improve their professional knowledge and skills, reduce workload, and improve work efficiency. This may effectively reduce pharmacists’ occupational stress. Hospitals, universities, clinical pharmacists, and other relevant parties must collaborate to eliminate barriers. Hospitals should first focus on enhancing their electronic pharmaceutical care management system and establishing a standard process for pharmaceutical information services. Secondly, hospitals should enhance the availability of qualified clinical pharmacists and allocate dedicated spaces for pharmaceutical care. It is crucial to ensure that clinical pharmacists have sufficient time to focus on pharmaceutical care and to pay attention to the standardized training of clinical pharmacists in relation to application skills. Thirdly, universities should consider integrating the computer network technology required by clinical pharmacists into their teaching and learning programs. Additionally, it is important to provide more opportunities for continuing education.

This study also has several limitations. First, the convenience sampling used in the stratified sampling procedure may have resulted in a biased sample. Second, the barriers of pharmaceutical care perceived by clinical physicist scale was newly developed for this study, and the version of the BJSQ scale used in the study was translated into Chinese. Although both have acceptable reliability within this sample, there may still be limitations before they are tested in a larger sample with formal validation analyses (19).

## 5 Conclusion

Pharmacists play a crucial role in treatment teams, and their performance significantly impacts patients’ treatment outcomes and safety. Identifying clinical pharmacists’ perceived work barriers can effectively reduce their occupational stress, improving their work efficiency and quality. This, in turn, gradually enhances the level of pharmaceutical care services. This study examines the relationship between perceived barriers of pharmaceutical care and occupational stress among clinical pharmacists in tertiary hospitals in China, using a nationwide survey. The results indicate that perceived barriers in the resource and self-improvement dimensions are significantly associated with increased occupational stress. Removing barriers perceived by pharmacists in terms of resources and self-improvement may effectively alleviate occupational stress on clinical pharmacists. The study also found that professional titles and specialty were associated with clinical pharmacists’ occupational stress. These findings could provide evidence to support the improvement of clinical pharmacy services in China and other developing countries for policy makers, the higher education system, clinical pharmacy training organizations, and hospital administrators.

## Data availability statement

The raw data supporting the conclusions of this article will be made available by the authors, without undue reservation.

## Ethics statement

The studies involving humans were approved by China Pharmaceutical University. The studies were conducted in accordance with the local legislation and institutional requirements. The participants provided their written informed consent to participate in this study.

## Author contributions

YZ: Writing – original draft. YH: Writing – review & editing. XX: Writing – review & editing.
