# Supplementary material for: Association of the barriers of pharmaceutical care perceived by clinical pharmacists and occupational stress in tertiary hospitals of China
Source: Front Public Health. 2024 Apr 9;12:1342565. doi: 10.3389/fpubh.2024.1342565 (PMC11035884; doi:10.3389/fpubh.2024.1342565)
Supplement: Supplementary file 1 [file Data_Sheet_1.docx]

**Association of the barriers of pharmaceutical care perceived by clinical pharmacists and occupational stress in tertiary hospitals of China**

**Appendix 1: Questionnaire**

**Table 1 Questionnaire** **for the** **barriers of pharmaceutical care perceived by clinical pharmacist and occupational stress**

| **Dear clinical pharmacist:**  This is a questionnaire about the barriers of pharmaceutical care perceived by clinical pharmacist. It aims to study the impact of various barriers on the occupational stress of clinical pharmacists, so as to provide information and suggestions for the relief of the occupational stress of clinical pharmacists and the improvement of quality of pharmaceutical care in China. The survey results do not contain any of your identity information, and the survey results will be kept strictly confidential and used only for academic research and not for any commercial purpose.  The questions in the questionnaire are all single-choice questions or blank questions if there is no (multiple choice). Please answer according to your actual situation and attitude. If you have any  questions about the requirements of the questionnaire, please feel free to ask the investigator. Thank you for your support and cooperation. | |
| --- | --- |
| **Section 1** |  |
| 1. What is your gender? | □Male □Female |
| 2. What is your age?？ | _____years old |
| 3. What is your current marriage situation? | □Unmarried □Married  □Other (Divorced, Widowed, etc.) |
| 4. How many years have you worked in your current or similar position? | _____years |
| 5. What is your technical title? | □Junior title □Intermediate title  □Deputy senior title □senior title |
| 6. What is your degree and major at each level? (Multiple choices) | □Anti-infectives □Cardiology □Respiratory Medicine □Gastroenterology □Nephrology □Oncology □Organ transplantation □Intensive care □Endocrinology □Neurology □Other |
| 7. What is the type of the hospital you work in? | □ General hospital □Specialized hospital  □Traditional Chinese Medicine Hospital □Other |
| Section 2 | |
| 8.Your recent working status： | |
| (1) You have to do an enormous amount of work. | □agree □somewhat agree □somewhat disagree □disagree |
| (2) You cannot complete all your work in the allotted time. | □agree □somewhat agree □somewhat disagree □disagree |
| (3) You have to work very hard. | □agree □somewhat agree □somewhat disagree □disagree |
| (4) You have to focus your attention quite a lot. | □agree □somewhat agree □somewhat disagree □disagree |
| (5) You do a difficult job that requires a high level of knowledge and skill. | □agree □somewhat agree □somewhat disagree □disagree |
| (6) You have to constantly think about your work during working hours. | □agree □somewhat agree □somewhat disagree □disagree |
| (7) You can work at your own pace. | □agree □somewhat agree □somewhat disagree □disagree |
| (8) You can decide the order in which you do your work and the way you do it. | □agree □somewhat agree □somewhat disagree □disagree |
| (9) You can reflect your own opinions on the workplace’s work strategy. | □agree □somewhat agree □somewhat disagree □disagree |
| (10) You can often communicate with supervisor. | □agree □somewhat agree □somewhat disagree □disagree |
| (11) You can often communicate with co-worker. | □agree □somewhat agree □somewhat disagree □disagree |
| (12) You can strongly rely on supervisor if you have some trouble. | □agree □somewhat agree □somewhat disagree □disagree |
| (13) You can strongly rely on co-worker if you have some trouble. | □agree □somewhat agree □somewhat disagree □disagree |
| (14) Your supervisor kindly spends his\her time on your private problem. | □agree □somewhat agree □somewhat disagree □disagree |
| (15) Your co-worker kindly spends his\her time on your private problem. | □agree □somewhat agree □somewhat disagree □disagree |
| (16) You have job satisfaction in your work. | □agree □somewhat agree □somewhat disagree □disagree |
| Section 3 | |
| 9. You have encountered the following barriers in your work： | |
| (1) The pharmaceutical care electronic management system (such as hospital management system and pharmaceutical care software) of your hospital is difficult to use | □yes □no |
| (2) Your hospital is not equipped with enough pharmaceutical workers | □yes □no |
| (3) Your hospital lacks pharmaceutical service rules and regulations | □yes □no |
| (4) Your hospital lacks a special place to provide pharmaceutical services | □yes □no |
| (5) You lack special time to provide pharmaceutical services | □yes □no |
| (6) Your hospital lacks the electronic information system and prescription evaluation system required for pharmaceutical services | □yes □no |
| (7) Your don’t have the standardized service process and relevant documents | □yes □no |
| (8) You do not think you are a "medical service provider" who directly provides medical services to patients | □yes □no |
| (9) You lack communication with doctors and their support | □yes □no |
| (10) You lack communication with other medical service workers and their support | □yes □no |
| (11) You lack communication with patients and their support | □yes □no |
| (12) You cannot obtain the patient's medical information, including medical records, prescriptions, test results, etc | □yes □no |
| (13) You cannot modify the patient's therapeutic schedule | □yes □no |
| (14) You lack opportunities for continuing education | □yes □no |
| (15) You lack time for continuing education | □yes □no |
| (16) Lack of support from the leaders of your hospital in providing pharmaceutical services | □yes □no |
| (17) Lack of support from the department leaders of your hospital in providing pharmaceutical services | □yes □no |
| (18) Lack of support from corresponding laws and systems in providing pharmaceutical services | □yes □no |

**Table 2 Glossary of abbreviations and complete items of barriers**

| **Complete item** | **Abbreviation** |
| --- | --- |
| (1) The pharmaceutical care electronic management system (such as hospital management system and pharmaceutical care software) of your hospital is difficult to use | Electronic systems are difficult to use |
| (2) Your hospital is not equipped with enough pharmaceutical workers | Staffing of pharmacy |
| (3) Your hospital lacks pharmaceutical service rules and regulations | Rules and regulations |
| (4) Your hospital lacks a special place to provide pharmaceutical services | Specific place |
| (5) You lack special time to provide pharmaceutical services | Specific time |
| (6) Your hospital lacks the electronic information system and prescription evaluation system required for pharmaceutical services | Lack of Electronic systems are difficult to uses |
| (7) Your don’t have the standardized service process and relevant documents | Standardized procedures and records |
| (8) You do not think you are a "medical service provider" who directly provides medical services to patients | Self-identified as non-direct health care provider |
| (9) You lack communication with doctors and their support | Physician’s communication and support |
| (10) You lack communication with other medical service workers and their support | Other staff’s support and communication |
| (11) You lack communication with patients and their support | Patient’s communication and support |
| (12) You cannot obtain the patient's medical information, including medical records, prescriptions, test results, etc | Patient's medical information |
| (13) You cannot modify the patient's therapeutic schedule | Modification of therapeutic schedule |
| (14) You lack opportunities for continuing education | Opportunities for continuing education |
| (15) You lack time for continuing education | Time for continuing education |
| (16) Lack of support from the leaders of your hospital in providing pharmaceutical services | Support from leaders of hospital |
| (17) Lack of support from the department leaders of your hospital in providing pharmaceutical services | Support from department leaders |
| (18) Lack of support from corresponding laws and systems in providing pharmaceutical services | Support from the legal system |
