# Supplementary material for: Association of the barriers of pharmaceutical care perceived by clinical pharmacists and occupational stress in tertiary hospitals of China
Source: Front Public Health. 2024 Apr 9;12:1342565. doi: 10.3389/fpubh.2024.1342565 (PMC11035884; doi:10.3389/fpubh.2024.1342565)
Supplement: Supplementary file 2 [file Data_Sheet_2.docx]

**Association of the barriers of pharmaceutical care perceived by clinical pharmacists and occupational stress in tertiary hospitals of China**

**Appendix 2: Questionnaire dimension reduction for the barriers of pharmaceutical care perceived by clinical pharmacists**

**Table 1 Results of questionnaire dimension reduction**

| **Items** |
| --- |
| **Resource** |
| Lack of Electronic systems are difficult to uses |
| Electronic systems are difficult to uses |
| Standardized procedures and records |
| Staffing of pharmacy |
| Specific place |
| Specific time |
| **Cooperation** |
| Physician’s communication and support |
| Other staff’s support and communication |
| Patient’s communication and support |
| **Leadership support and pharmacist’s right** |
| Support from leaders of hospitals |
| Support from department leaders |
| Support from the legal system |
| Patient's medical information |
| **Self-improvement** |
| Opportunities for continuing education |
| Time for continuing education |

**Table 2 Items that should be deleted based on exploratory factor analysis (EFA)**

| Items |
| --- |
| Rules and regulations |
| Self-identified as non-direct health care provider |
| Modification of therapeutic schedule |

**Results of exploratory factor analysis (EFA)**

| **KMO and Bartlett's Test** | | |
| --- | --- | --- |
| Kaiser-Meyer-Olkin Measure of Sampling Adequacy. | | .888 |
| Bartlett's Test of Sphericity | Approx. Chi-Square | 3744.542 |
|  | df | 153 |
|  | Sig. | .000 |

| **Communalities** | | |
| --- | --- | --- |
|  | Initial | Extraction |
| Electronic systems are difficult to use | 1.000 | .507 |
| Staffing of pharmacy | 1.000 | .465 |
| Rules and regulations | 1.000 | .440 |
| Specific place | 1.000 | .490 |
| Specific time | 1.000 | .513 |
| Lack of Electronic systems are difficult to uses | 1.000 | .592 |
| Standardized procedures and records | 1.000 | .581 |
| Self-identified as non-direct health care provider | 1.000 | .310 |
| Physician’s communication and support | 1.000 | .758 |
| Other staff’s support and communication | 1.000 | .728 |
| Patient’s communication and support | 1.000 | .663 |
| Patient's medical information | 1.000 | .376 |
| Modification of therapeutic schedule | 1.000 | .297 |
| Opportunities for continuing education | 1.000 | .696 |
| Time for continuing education | 1.000 | .786 |
| Support from leaders of hospitals | 1.000 | .675 |
| Support from department leaders | 1.000 | .691 |
| Support from the legal system | 1.000 | .496 |
| Extraction Method: Principal Component Analysis. | | |

| **Total Variance Explained** | | | | | | | | | |
| --- | --- | --- | --- | --- | --- | --- | --- | --- | --- |
| Component | Initial Eigenvalues | | | Extraction Sums of Squared Loadings | | | Rotation Sums of Squared Loadings | | |
|  | Total | % of Variance | Cumulative % | Total | % of Variance | Cumulative % | Total | % of Variance | Cumulative % |
| 1 | 5.930 | 32.945 | 32.945 | 5.930 | 32.945 | 32.945 | 3.311 | 18.397 | 18.397 |
| 2 | 1.691 | 9.395 | 42.340 | 1.691 | 9.395 | 42.340 | 2.732 | 15.179 | 33.576 |
| 3 | 1.320 | 7.334 | 49.674 | 1.320 | 7.334 | 49.674 | 2.387 | 13.263 | 46.839 |
| 4 | 1.123 | 6.240 | 55.914 | 1.123 | 6.240 | 55.914 | 1.633 | 9.075 | 55.914 |
| 5 | .895 | 4.973 | 60.887 |  |  |  |  |  |  |
| 6 | .841 | 4.670 | 65.557 |  |  |  |  |  |  |
| 7 | .766 | 4.258 | 69.815 |  |  |  |  |  |  |
| 8 | .729 | 4.049 | 73.863 |  |  |  |  |  |  |
| 9 | .696 | 3.868 | 77.731 |  |  |  |  |  |  |
| 10 | .604 | 3.358 | 81.089 |  |  |  |  |  |  |
| 11 | .543 | 3.014 | 84.103 |  |  |  |  |  |  |
| 12 | .530 | 2.945 | 87.048 |  |  |  |  |  |  |
| 13 | .485 | 2.695 | 89.743 |  |  |  |  |  |  |
| 14 | .449 | 2.493 | 92.236 |  |  |  |  |  |  |
| 15 | .409 | 2.270 | 94.506 |  |  |  |  |  |  |
| 16 | .389 | 2.160 | 96.666 |  |  |  |  |  |  |
| 17 | .338 | 1.876 | 98.542 |  |  |  |  |  |  |
| 18 | .262 | 1.458 | 100.000 |  |  |  |  |  |  |
| Extraction Method: Principal Component Analysis. | | | | | | | | | |

| **Component Matrix^a^** | | | | |
| --- | --- | --- | --- | --- |
|  | Component | | | |
|  | 1 | 2 | 3 | 4 |
| Specific time | .673 |  |  |  |
| Support from leaders of hospitals | .672 |  |  |  |
| Standardized procedures and records | .666 |  |  |  |
| Specific place | .654 |  |  |  |
| Other staff’s support and communication | .631 |  |  |  |
| Rules and regulations | .626 |  |  |  |
| Physician’s communication and support | .614 |  |  |  |
| Patient’s communication and support | .589 |  |  |  |
| Lack of Electronic systems are difficult to uses | .581 |  |  |  |
| Support from department leaders | .580 |  |  |  |
| Support from the legal system | .580 |  |  |  |
| Staffing of pharmacy | .530 |  |  |  |
| Modification of therapeutic schedule | .519 |  |  |  |
| Opportunities for continuing education | .518 |  |  |  |
| Electronic systems are difficult to use |  |  |  |  |
| Self-identified as non-direct health care provider |  |  |  |  |
| Patient's medical information |  |  |  |  |
| Time for continuing education |  |  |  | .631 |
| Extraction Method: Principal Component Analysis. | | | | |
| a. 4 components extracted. | | | | |

| **Rotated Component Matrix^a^** | | | | |
| --- | --- | --- | --- | --- |
|  | Component | | | |
|  | 1 | 2 | 3 | 4 |
| Lack of Electronic systems are difficult to uses | .755 |  |  |  |
| Electronic systems are difficult to use | .709 |  |  |  |
| Standardized procedures and records | .687 |  |  |  |
| Staffing of pharmacy | .657 |  |  |  |
| Specific place | .605 |  |  |  |
| Specific time | .540 |  |  |  |
| Rules and regulations |  |  |  |  |
| Physician’s communication and support |  | .845 |  |  |
| Other staff’s support and communication |  | .815 |  |  |
| Patient’s communication and support |  | .779 |  |  |
| Self-identified as non-direct health care provider |  |  |  |  |
| Modification of therapeutic schedule |  |  |  |  |
| Support from department leaders |  |  | .797 |  |
| Support from leaders of hospitals |  |  | .741 |  |
| Support from the legal system |  |  | .569 |  |
| Patient's medical information |  |  | .541 |  |
| Time for continuing education |  |  |  | .866 |
| Opportunities for continuing education |  |  |  | .773 |
| Extraction Method: Principal Component Analysis.  Rotation Method: Varimax with Kaiser Normalization. | | | | |
| a. Rotation converged in 6 iterations. | | | | |

| **Component Transformation Matrix** | | | | |
| --- | --- | --- | --- | --- |
| Component | 1 | 2 | 3 | 4 |
| 1 | .629 | .516 | .497 | .300 |
| 2 | -.680 | .704 | .006 | .204 |
| 3 | -.340 | -.483 | .619 | .518 |
| 4 | .162 | -.063 | -.608 | .775 |
| Extraction Method: Principal Component Analysis.  Rotation Method: Varimax with Kaiser Normalization. | | | | |

**Results of confirmatory factor analysis (CFA)**


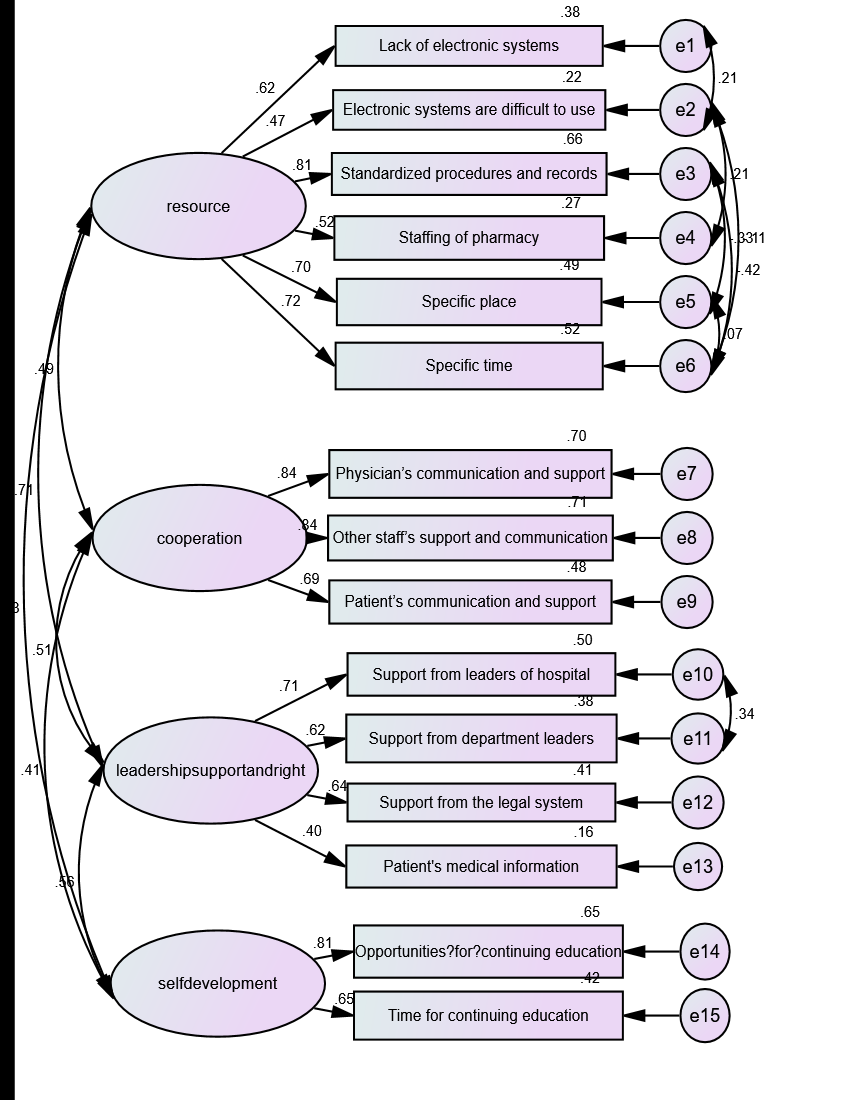


**CFA Model（MI)**

**Overall fitting coefficient table**

| χ^2^/df | RMSEA | GFI | AGFI | CFI | IFI | TLI |
| --- | --- | --- | --- | --- | --- | --- |
| 1.983 | 0.040 | 0.969 | 0.951 | 0.975 | 0.976 | 0.967 |

χ^2^/df=1.983<3，RMSEA=0.04<0.05，GFI=0.969>0.9，AGFI=0.951>0.9，CFI=0.975>0.9，IFI=0.976>0.9，TFI=0.967>0.9，the model fitting index is good.
